# Genomic Selection for Lodging-Related Traits in Double-Cropping Rice

**DOI:** 10.3390/plants15050785

**Published:** 2026-03-04

**Authors:** Wenyu Lu, Jicheng Yue, Jinzhao Liu, Xilong Yuan, Hui Wang, Tao Guo, Hong Liu

**Affiliations:** 1 College of Agriculture, South China Agricultural University, Guangzhou 510642, China; spacebreedinglwy@stu.scau.edu.cn (W.L.);; 2 Modern Agricultural Development Service Center of Zhongyang County, Lvliang 033400, China

**Keywords:** double cropping rice, lodging resistance, genomic selection, breeding

## Abstract

Genomic selection (GS) is a promising tool to accelerate genetic gain for complex traits. In this study, we evaluated the potential of GS for the improvement of seven lodging-related traits in double-cropping rice in Southern China using 438 rice accessions. The traits examined included the length and bending resistance of the third and fourth internodes (IL3, IL4, BR3, BR4), plant height (PH), and the ratio of internode length to plant height (IL3/PH, IL4/PH). Significant phenotypic differences were observed for all traits between the two seasons. In comparisons of cross-validation and independent prediction, GBLUP and BayesLASSO outperformed LightGBM across all traits in both seasons. Across all evaluated traits, prediction accuracies (Pearson’s *r*) ranged from 0.33 to 0.78 in cross-validation and from 0.28 to 0.75 in independent prediction using the GBLUP model. Bending resistance exhibited lower prediction accuracy due to its lower genomic heritability. Correlation analysis revealed that plant height was not significantly correlated with culm bending resistance, suggesting that these traits are genetically independent. We utilized GBLUP models trained on our experimental data to predict the genomic estimated breeding values (GEBVs) of the 3000 Rice Genomes Project (3kRG) dataset. The results demonstrated that GS can efficiently enrich the proportion of highly lodging-resistant accessions, increasing it from 31.40% in the base 3kRG population to a maximum of 83.00% among the top 200 selected individuals. Furthermore, indirect selection for traits with higher heritability, such as IL and IL/PH, was more effective at screening highly lodging-resistant cultivars than direct selection for BR. Our research demonstrates the feasibility of applying genomic selection for the breeding of lodging-resistant varieties in double-cropping rice and provides a foundation for further applications.

## 1. Introduction

Lodging primarily occurs during the late grain-filling stage of rice, negatively impacting yield; a 2% field lodging rate leads to a 1% reduction in total production [1]. Plant height and basal internode length are critical traits in rice architecture, directly impacting lodging resistance and yield [2,3]. The South China double-cropping rice region is a primary production area, contributing 12.3% to the total national rice output. In this system, rice lodging is a frequent occurrence driven by a combination of adverse weather conditions, pest and disease infestations, as well as improper fertilization and suboptimal cultivation practices [4,5]. The recessive *sd1* allele was first introduced into rice breeding to develop semi-dwarf varieties [6] and was subsequently widely distributed across Asia to enhance lodging resistance [7]. A strategy solely focused on reducing plant height and basal internode length is restrictive, as rice plants cannot become excessively stunted. Rice breeding experts suggest that appropriately increasing plant height can boost rice yield, but this may concurrently increase the risk of lodging [8].

Enhancing rice stem strength is a direct and critical pathway to increasing the plant’s lodging resistance [9]. For instance, the *Strong Culm* genes *SCM2* (identical to *APO1*) and *SCM3* (identical to *OsTB1*) have been identified as key regulators that enhance culm strength by increasing culm diameter and the number of large vascular bundles [9,10]. While their effects are additive and have successfully improved the lodging resistance of Japonica rice, they have not yet been widely utilized in Indica rice breeding practices.

Until now, MAS has played an essential role in rice breeding, facilitating the rapid development of varieties with enhanced biotic (e.g., blast, bacterial leaf blight) and abiotic (e.g., submergence, salinity) stress tolerances [11,12,13,14]. However, MAS is primarily limited to the introgression of major genes or large-effect quantitative trait loci (QTLs). For complex quantitative traits governed by a large number of small-effect QTLs, genomic selection (GS) offers a superior alternative by predicting individual genomic estimated breeding values (GEBVs) through genome-wide markers [15,16]. First proposed in animal breeding [17], GS has recently become a cornerstone methodology for enhancing breeding efficiency across various crops due to reduced genotyping costs [18].

Genomic selection (GS) models are commonly categorized into two broad classes: (i) parametric approaches (e.g., GBLUP and Bayesian models), which rely on explicit distributional assumptions and typically employ linear models to capture genotype–phenotype associations, demonstrating proven efficacy; and (ii) non-parametric methods (e.g., random forests and kernel-based approaches), which operate without predefined genetic architectures and predict genomic estimated breeding values (GEBVs) through complex nonlinear modeling frameworks [19,20]. As an agronomic trait observed in the reproductive stage of rice, the slow progress in breeding for lodging resistance is due to its lengthy investigation cycle. The lodging resistance needs to be tested in the reproductive stage, which requires large-scale, high-cost field sampling operations in the late period of the growing season. This research aims to test and verify the practicability of applying GS methods to lodging-related traits to provide an efficient genomic-based approach to enhance rice lodging resistance.

The present study was designed to systematically evaluate the potential of genomic selection (GS) for improving lodging resistance in a double-cropping rice system. The specific objectives were: (1) to characterize the phenotypic variation, heritability, and correlations of seven key lodging-related traits (including internode lengths IL3/IL4, bending resistance BR3/BR4, their ratios to plant height, and plant height itself) across two distinct growing seasons; (2) to compare the prediction accuracy of contrasting GS models, namely parametric (GBLUP, BayesLASSO) and non-parametric (LightGBM) approaches, through both cross-validation and independent prediction; and (3) to assess the practical utility and generalization capability of our models by predicting breeding values for lodging resistance in the large-scale 3000 Rice Genomes Project dataset.

## 2. Result

### 2.1. Phenotypic Characterization and Trait Correlation Analysis Across Two Seasons

**Figure 1:**
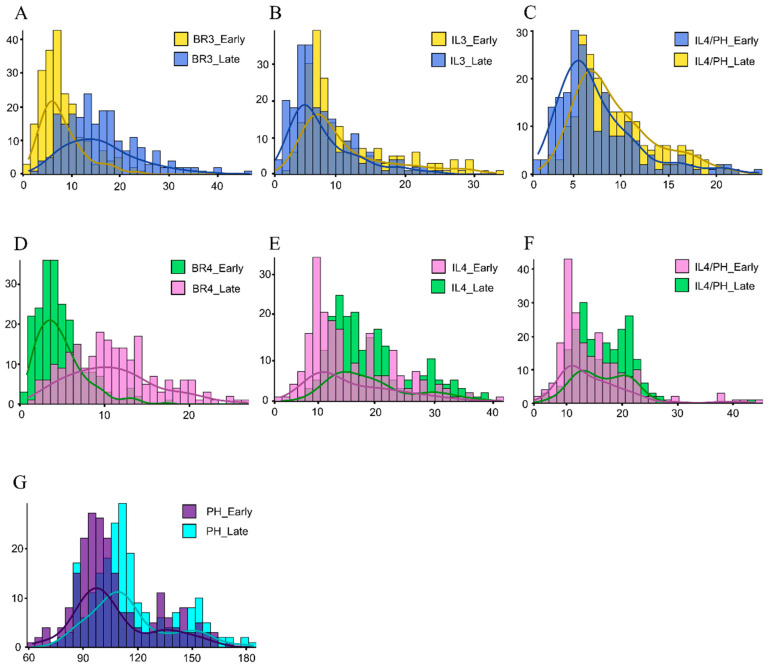
Frequency distribution histogram for two seasons. (**A**): Bending resistance of the third internode (BR3, in Newtons). (**B**): Internode length of the third internode (IL3, in cm). (**C**): The ratio of third internode length to plant height (IL3/PH). (**D**): Bending resistance of the fourth internode (BR4, in Newtons). (**E**): Internode length of the fourth internode (IL4, in cm). (**F**): The ratio of fourth internode length to plant height (IL4/PH). (**G**): Plant height (PH, in cm).

To characterize the phenotypic variation across different growing environments, we evaluated seven lodging-related traits in both the early (ES) and late (LS) seasons (Table 1). Significant phenotypic divergence was observed for all evaluated traits (*p* < 0.0001). In the late season, rice accessions developed a more lodging-resistant ideotype, characterized by shorter plant height (mean = 107.70 cm vs. 116.30 cm) and reduced basal internode length (e.g., IL3 dropped from 11.23 cm to 8.13 cm) (Figure 1). Conversely, the biomechanical strength of the culms was significantly enhanced in the late season, with BR3 and BR4 increasing by 99.5% and 126.6% compared to the early season, respectively. These results indicate that the late-season environment (characterized by shortening days) effectively restricts longitudinal growth while promoting the accumulation of culm strength.

**Figure 2:**
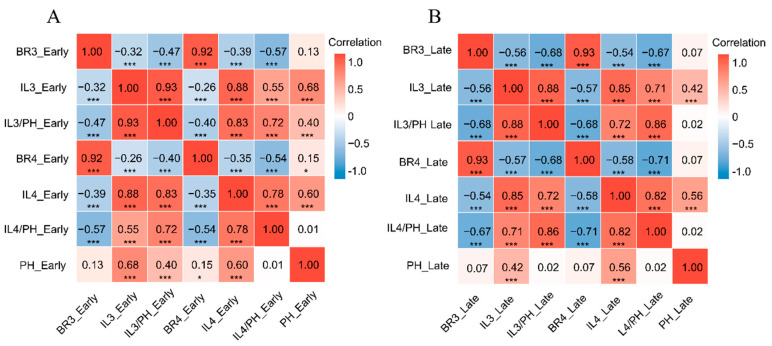
Correlation analysis heatmap. (**A**): Correlation heatmap for the early season; (**B**): Correlation heatmap for the late season. The x- and y-axes display the seven traits evaluated in this study: PH, plant height; IL3 and IL4, length of the third and fourth basal internodes, respectively; BR3 and BR4, culm bending resistance of the third and fourth basal internodes, respectively; IL3/PH and IL4/PH, the ratio of internode length to plant height for the third and fourth internodes, respectively. The numerical values within each cell represent the Pearson’s correlation coefficient (r). The color gradient on the right indicates the strength of the correlation, ranging from −1.0 (dark blue, perfect negative correlation) to 1.0 (dark red, perfect positive correlation). Asterisks denote statistical significance: * *p* < 0.05, *** *p* < 0.0001.

As shown in Figure 2, correlation analysis revealed consistent relationships across seasons. The bending resistances of the third and fourth internodes (BR3, BR4) were strongly positively correlated with each other (r > 0.92). Conversely, bending resistance was significantly negatively correlated with internode length (IL) and the IL/PH ratio. Plant height (PH) was positively correlated with IL. Critically, the correlation between PH and bending resistance was negligible and non-significant in both seasons.

### 2.2. Prediction Accuracy and Genomic Heritability Analysis

**Figure 3:**
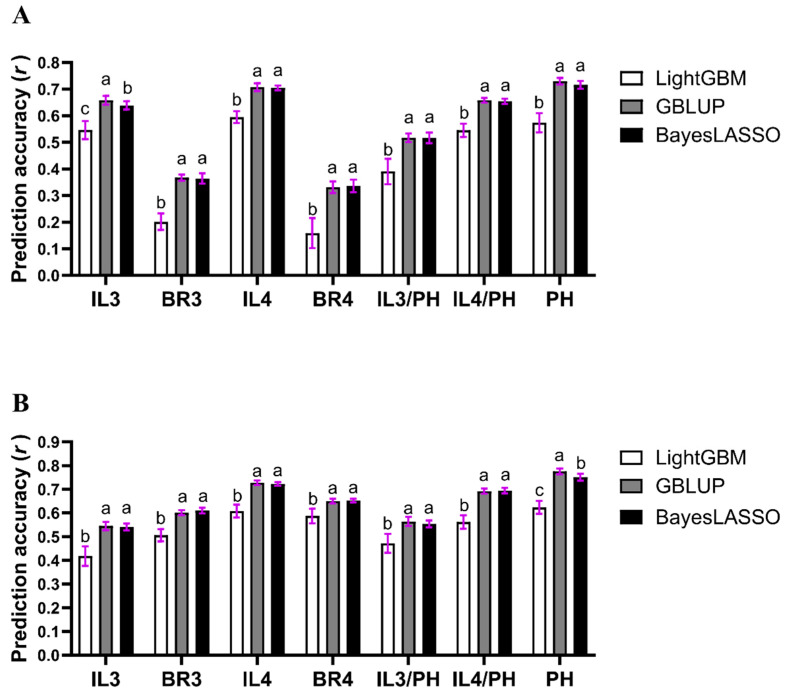
Comparison of prediction accuracy (Pearson’s *r*) across three genomic selection models in two growing seasons. (**A**) Prediction accuracy for traits in the early season. (**B**) Prediction accuracy for traits in the late season. Models included LightGBM (white bars), GBLUP (gray bars), and BayesLASSO (black bars). The purple error bars denote the standard deviation (SD) calculated from 20 independent repetitions of 5-fold cross-validation, indicating the stability of model performance. Traits examined: IL3 and IL4, length of the third and fourth basal internodes; BR3 and BR4, culm bending resistance; IL3/PH and IL4/PH, the ratio of internode length to plant height; and PH, plant height. Error bars represent the standard deviation across 20 independent repetitions of 5-fold cross-validation. For each trait, values followed by different lowercase letters (a, b, c) indicate significant differences among the three GS models within the same growing season (*p* < 0.05), as determined by Tukey’s multiple comparison test.

To evaluate the genetic basis and prediction performance for the seven lodging-related traits, we estimated their genomic heritabilities ($h_{g}^{2}$) and evaluated the prediction accuracies (r) of the three GS models (Table 2; Figure 3), using 20 repetitions of 5-fold cross-validation within the core set of 217 accessions.

Genomic heritability varied substantially across the traits, with plant height (PH) showing the highest ($h_{g}^{2}$ = 0.7687), followed by the fourth and third internode lengths (IL4: 0.6257; IL3: 0.5054). In contrast, culm bending resistance and ratio-related traits exhibited lower genomic heritabilities, with BR4 being the lowest ($h_{g}^{2}$ = 0.2297) (Table 2). Across all models and seasons, our results demonstrated a strong positive association between genomic heritability and prediction accuracy, where traits with higher $h_{g}^{2}$ generally yielded superior prediction performance. In this study, superior performance is characterized by the combination of high mean correlation coefficients (accuracy) and high stability, the latter of which is indicated by lower standard deviations across repetitions.

To statistically compare these accuracies, we conducted a two-way analysis of variance (ANOVA) for each trait, treating “GS models” and “growing seasons” as the two main factors (Table S4). The ANOVA revealed that the main effects of both the GS model and the growing season were highly significant for all traits (*p* < 0.0001). Furthermore, a significant interaction effect (GS model × season) was observed for five of the seven traits (IL3, BR3, BR4, IL3/PH, and IL4/PH), indicating that the magnitude of the accuracy differences between models varied depending on the season. Nevertheless, post hoc multiple comparison tests within each season confirmed a consistent trend: the parametric models (GBLUP and BayesLASSO) significantly outperformed the non-parametric LightGBM model across both environments (Figure 3).

For high-heritability traits like PH and IL4, the prediction accuracies remained high and consistent across the seasons (average *r* > 0.65 for parametric models). However, traits with lower heritability (BR3, BR4, and ratio traits) displayed a distinct seasonal bias, performing significantly better in the late season. For instance, the accuracy for BR4 using LightGBM surged from 0.1592 in the early season to 0.5873 in the late season (Table 2), suggesting that environmental conditions in the late season may favor the expression of genetic variance for culm strength.

The GBLUP and BayesLASSO models not only achieved higher mean prediction accuracies but also exhibited high stability across repetitions, as evidenced by the relatively small standard deviations (represented by the purple error bars in Figure 3). This consistent performance suggests that the parametric models are less sensitive to random variations in training set partitioning for lodging-related traits.

### 2.3. Independent Prediction of Lodging-Related Phenotypes

To evaluate the generalization ability of the GS models to novel genotypes and environments, we conducted independent seasonal predictions. The 217 accessions common to both seasons served as the training set, while the accessions unique to each season (98 in the early season and 123 in the late season) were utilized as independent testing sets (Table 3).

**Figure 4:**
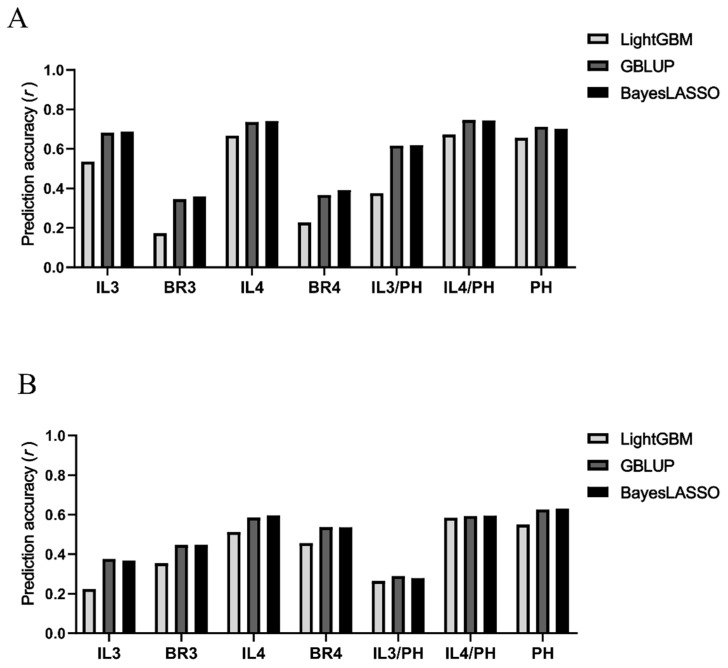
Independent prediction for two seasons in three methods. (**A**): Independent prediction for early season. (**B**): Independent prediction for late season.

Consistent with the cross-validation results, the independent prediction accuracies (r) were strongly associated with the genomic heritabilties ($h_{g}^{2}$) of the traits. Plant height (PH) consistenty yielded the highest accuracies across all models, ranging from 0.5502 (LigntGBM, late season) to 0.7126 (GBLUP, early season), with minimal fluctuation (SD = 0.0590), indicating high predictive robustness (Figure 4).

The predictive performance for internode length (IL) and bending resistance (BR) exhibited distinct seasonal patterns. IL traits showed superior accuracy in the early season (e.g., IL4 r ranged from 0.6683 to 0.7422), whereas IL3 accuracy decined in the late season, with a maximum r of only 0.3762 for GBLUP. Conversely, the prediction accuracies for bending resistance were higher in the late season (e.g., BR4 r ranged from 0.4560 to 0.5375), with BR4 consistently outperforming BR3 across both seasons. Regarding the internode length to plant height ratios, 1L4/PH maintained acceptable accuracy, which was higher in the early season (mean r = 0.7217) than in the late season (mean r = 0.5909). In contrast, L3/PH demonstrated poor stability across models and seasons (SD = 0.1676) (Figure 4).

Overall, the parametric models (GBLUP and BayesLASSO) demonstrated superior predictive power compared to the non-parametric LightGBM model for most lodging-related traits. Furthermore, traits associated with the third internode generally exhibited higher fluctuations in prediction accuracy than those of the fourth internode, suggesting greater environmental sensitivity or lower genetic stability in the upper basal internodes.

### 2.4. Cross-Population Prediction and Environment: Lodging Resistance Prediction of 3000 Rice Genomes Project

The genotype and phenotype data of all 438 accessions (315 in the early season, 340 in the late season) were used as the training set. Predicted values for the seven lodging-related traits of 2038 rice cultivars in the 3000 Rice Genomes Project were calculated for both seasons using GBLUP. In the phenotype dictionary of the 3000 Rice Genomes Project, “cust_repro” refers to culm strength at the reproductive stage and is divided into nine levels, from level 1 (no lodging) to level 9 (all plants lodged flat) (Table 4).

**Figure 5:**
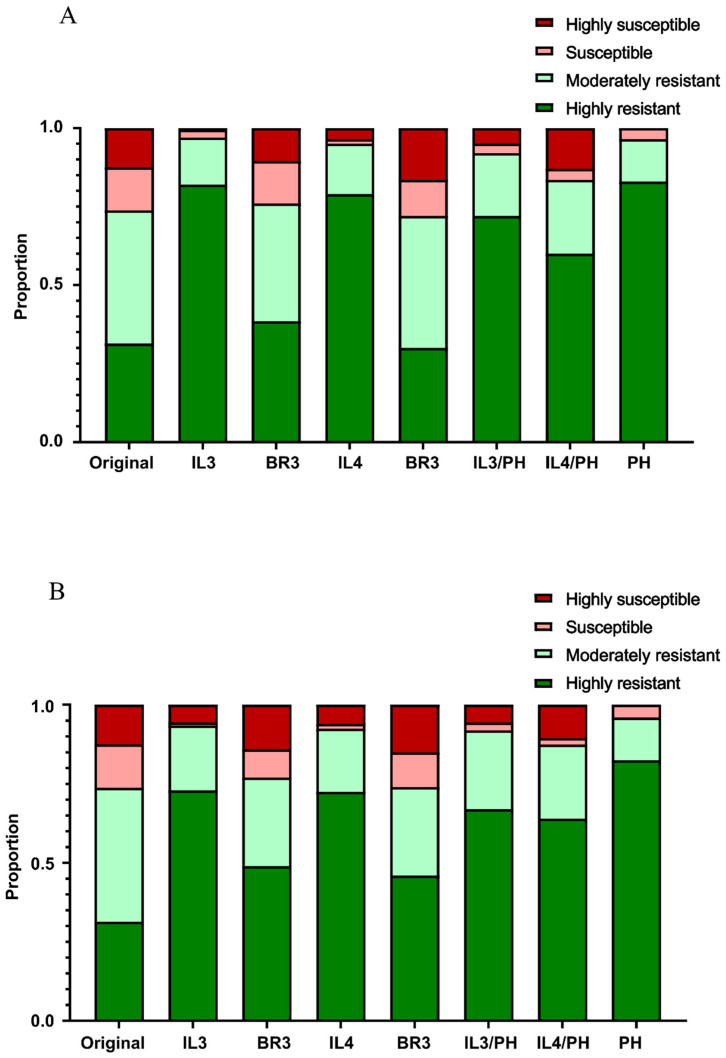
Proportion change in the lodging resistance levels in the top 200 individuals of 3kRG selected by GBLUP genomic selection. (**A**): Cross-population prediction using early season’s phenotype as train set. (**B**): Cross-population prediction using late season’s phenotype as train set.

In the original 3kRG dataset of 2038 cultivars, the proportions of accessions classified as highly lodging-resistant (HLR), moderately lodging-resistant (MLR), susceptible, and extremely susceptible were 31.40%, 42.39%, 13.74%, and 12.46%, respectively. Selection of the top 200 individuals based on the predicted genomic estimated breeding values (GEBVs) for any trait resulted in a marked enrichment of the combined HLR and MLR categories (Figure 5).

The efficiency of this selection varied across traits, but no significant differences were observed between models trained on the early versus late season data.

Indirect selection based on internode length (IL), plant height (PH), and their ratios (IL/PH) proved more effective than direct selection based on bending resistance (BR). Specifically, selection based on predicted PH effectively eliminated nearly all “susceptible” and “extremely susceptible” cultivars. Selection based on predicted IL or IL/PH further increased the combined proportion of HLR and MLR categories to between 83.5% (IL4/PH, late season) and 97% (IL3, early season). In contrast, selection based on BR traits provided only a marginal increase in the HLR proportion, with BR3 and BR4 yielding increases of 7.10–17.60% and 14.60%, respectively, across seasons (excluding BR4 in the early season). These results suggest that highly heritable proxy traits may be more reliable than direct culm strength measurements for identifying lodging resistance in large-scale populations.

## 3. Discussion

### 3.1. Genomic Selection for Lodging-Related Traits Should Be Done in the Early or Late Season for Their Respective Varieties

A primary discovery of this study is the profound phenotypic divergence of lodging-related traits between the early and late growing seasons, as evidenced by the highly significant differences in all seven traits (Table 1). Rice plants grown in the late season developed a more lodging-resistant phenotype, characterized by shorter, more compact statures (reduced PH, IL3, and IL4) but significantly stronger basal internodes (increased BR3 and BR4). This is similar to the conclusions drawn from previous studies on lodging resistance in barley and rice [3,21]. These results highlight the substantial impact of the growing environment on the expression of lodging-related traits.

This phenotypic divergence is hypothesized to be driven by the differential perception and response of rice to photoperiodic cues and internal circadian rhythms. As a typical short-day plant (SDP), photosensitive rice varieties require short-day induction to trigger the transition from vegetative to reproductive growth. In Guangzhou, the geographic location of this study, the early season (March to July) is characterized by progressively lengthening days until the summer solstice, which is unfavorable for short-day induction. Consistently, previous studies have demonstrated that photoperiod-sensitive rice accessions exhibit significantly reduced plant height when cultivated under short-day conditions [22]. Furthermore, specific genes regulating maturity have been reported to decrease culm length by accelerating the heading process under short-day environments [23]. In contrast, the late season (July to November) features continuously shortening days. These decreasing photoperiod conditions more readily induce rice to cease vegetative growth and enter the reproductive phase, thereby terminating the elongation of basal internodes earlier. Consequently, the lack of timely short-day induction in the early season results in prolonged internode elongation, leading to longer basal internodes, poorer bending resistance, and a higher ratio of internode length to plant height.

This finding demonstrates that the performance of lodging-related traits in the double-cropping rice of Southern China has a strong association with the planting season. This implies that a universal strategy cannot be applied to breeding for lodging resistance in rice varieties adapted for early and late seasons. To effectively improve the lodging resistance of double-cropping rice, distinct genomic selection models should be developed for rice adapted to different seasons. The phenotype and genotype data for both the early and late seasons provided by this study can serve as a training set for genomic selection aimed at improving lodging resistance in double-cropping rice.

### 3.2. Decoupling Plant Height and Culm Strength Could Be a New Strategy in Rice Breeding

A consistent finding from our correlation analysis (Figure 2) across both seasons was the revelation of a critical insight: while confirming the expected negative correlation between internode length and bending strength, we found that plant height (PH) was only weakly and non-significantly correlated with culm bending resistance (BR) and the internode length to plant height ratio (IL/PH). This statistical independence strongly suggests that the genetic loci controlling overall plant stature may be distinct from those governing the biomechanical strength of the culm tissue. This finding challenges the conventional breeding paradigm that, since the Green Revolution, has primarily relied on reducing plant height to increase the harvest index as the main strategy against lodging [24]. Indeed, emerging evidence indicates that reducing plant height can compromise the source–sink balance, thereby leading to a decline in yield potential [25]. Corroborating this, a recent study of 578 accessions from the 3k Rice Genomes Project demonstrated that grain yield per plant increased linearly with plant height, reaching an optimum at 118 cm [26]. Collectively, these findings illuminate a promising alternative pathway for sd1-dependent semi-dwarf breeding: breeders can pursue a “tall-but-strong” ideal architecture by simultaneously selecting for increased plant height and enhanced culm strength. This dual-pronged selection strategy offers a viable route to break the longstanding dilemma between yield potential and lodging resistance.

### 3.3. Superiority of Parametric Models for Lodging-Related Traits and the Influence of Genomic Heritability

The comparison of the GS models demonstrated that the parametric methods (GBLUP and BayesLASSO) consistently outperformed the non-parametric approach (LightGBM) in both cross-validation and independent prediction. This indicates that the genetic architecture of the evaluated lodging traits is predominantly additive and polygenic, governed by numerous small-effect loci rather than a few predominant QTLs. GBLUP, which assumes a normal distribution of marker effects, is theoretically well-suited for such an infinitesimal model. The observation that BayesLASSO—which allows for differential shrinkage of marker effects—did not outperform GBLUP suggests a lack of major-effect QTLs in this population [27]. Furthermore, the inferior performance of LightGBM can be attributed to the fact that nonlinear interaction effects (e.g., epistasis) do not constitute a major component of the phenotypic variance. Additionally, the relatively small sample size (N ≈ 200–350) likely hindered the non-parametric model from learning complex patterns without overfitting, a common challenge known as the “curse of dimensionality.” Overall, prediction accuracy remained highly contingent on genomic heritability [28]. Traits with high genomic heritability, like PH (0.77) and IL4 (0.63), yielded robust prediction accuracies, often exceeding 0.70. Conversely, the lower-genomic-heritability bending-resistance traits (BR3 and BR4) showed more modest and variable accuracies. Notably, the predictability of these culm-strength-related traits was markedly higher in the late season, which may be attributed to environmental conditions that maximize the expression of genetic variance for culm strength.

### 3.4. Application of Genomic Selection in Large-Scale Screening and Elite Parent Discovery

The application of our GBLUP model to the 3000 Rice Genomes Project (3kRG) serves as a powerful proof-of-concept for large-scale germplasm mining. We demonstrate that GS can efficiently eliminate susceptible germplasm at early stages, provided genotyping remains more cost-effective than phenotyping. A particularly insightful finding was that indirect selection based on highly heritable proxy traits (PH, IL3, IL4, and their ratios) was significantly more effective at identifying lodging-resistant individuals than direct selection for the lower-heritability bending-resistance traits. For instance, selecting for shorter IL3 successfully enriched the proportion of resistant accessions to 97%, compared to the marginal gains achieved through direct selection on BR. This underscores a strategic shift for breeders: for complex biomechanical traits that are difficult to phenotype or possess low heritability, targeting correlated, easily measurable traits with stronger genetic signals can yield greater and more efficient genetic gains. Based on our results, we recommend the GBLUP model for such applications due to its computational efficiency and robust accuracy across diverse populations.

Beyond large-scale genomic prediction, identifying specific elite donors within the existing population is crucial for immediate breeding applications. As detailed in Table S2, we identified the top 20 accessions with the highest bending resistance (BR3) in both the early (Table S2A) and late seasons (Table S2B). Notably, four accessions—R467 (IR 78875-176-B-1-B), R145 (Wai 128), R533 (Guanghong 1), and R309 (Huangruanxiuzhan)—consistently ranked among the top performers in both environments (highlighted in orange). These stable genotypes, primarily Indica breeding lines from South China, represent ideal parental candidates for lodging-resistance breeding, as their superior culm strength appears less sensitive to seasonal environmental fluctuations. The inclusion of both modern improved cultivars (e.g., R309) and traditional landraces (e.g., R533) in this elite group suggests that while modern breeding has successfully incorporated culm strength, local landraces remain vital reservoirs of unique genetic diversity for further enhancing the biomechanical resilience of rice stalks.

## 4. Materials and Methods

### 4.1. Plant Materials

The natural population utilized in this study consisted of 438 unique rice accessions. Due to factors such as asynchronous development and plant loss, complete phenotypic data were successfully obtained for 315 accessions in the early season and 340 accessions in the late season. A core set of 217 accessions produced complete data across both seasons and was used for comparative analyses. The 438 rice accessions utilized in this study originated from diverse geographic regions, with Guangdong Province, China, serving as the primary source. Specifically, the population comprised 181 accessions from Guangdong (41.3%), 66 from Japan (15.1%), and 29 from the Philippines (6.6%). Additionally, 46 accessions (10.5%) were sourced from other provinces within the South China rice region, including Guangxi, Fujian, Hunan, and Hainan, while the remaining accessions originated from Vietnam and other countries.

The field experiment was conducted in 2022 at the Zengcheng Teaching and Research Base of South China Agricultural University (SCAU) in Guangzhou, China. For the early season, the seeds were sown in March and the seedlings were transplanted in April. For the late season, sowing occurred in early August followed by transplanting in late August. Each accession was planted in a single plot. The plot design consisted of 40 plants arranged in eight rows, with five plants per row. A spacing of 20 cm was maintained both between rows and between plants within each row, resulting in a plot of 1.4 m in length and 0.8 m in width. To prevent interference and facilitate field management, adjacent plots were separated by a 40 cm wide walkway. Standard local cultivation and management practices were uniformly applied to all plots in both seasons.

### 4.2. Field Phenotyping

The heading date for each accession was recorded when 50% of the plants had visible panicles. Phenotypic sampling was conducted 25 days after the heading date. For each plot, three representative plants were selected from the inner rows to avoid border effects. The main tiller (the tiller bearing the main panicle) of each selected plant was carefully uprooted. Plant height was first measured on the intact tiller, from the base to the tip of the panicle (excluding the awn, in cm). Subsequently, the tiller was washed to remove any soil, and the leaf sheaths were manually peeled away to expose the culm. The third and fourth basal internodes were then precisely excised using scissors. The length of each isolated internode was measured first (in cm). Immediately following the length measurement, the same internode samples were used for the bending resistance test (in Newtons, N).

The bending resistance was measured with the help of a culm strength tester (Zhejiang Topu Yunnong Technology Co., Ltd., Model YYD-1A). Referring to the method described in Ookawa’s study, each internode was positioned between a pair of support points and the hitting point was set to the middle of the internode [29]. The peak breaking force was recorded with the culm strength tester during stem bending upon drawing the pulling lever at a constant rate. The peak breaking force was recorded as the bending resistance of the internode.

The phenotypes of the 3000 Rice Genomes Project were downloaded from the Rice SNP-seek Database [30].

### 4.3. Whole-Genome Sequencing and Variant Detection

Library construction was performed using the NEB Next^®^ Ultra DNA Library Prep Kit (New England Biolabs, USA). Paired-end whole-genome sequencing (PE150) was conducted on an Illumina NovaSeq (Illumina, Inc., San Diego, CA, USA) platform with 10× coverage depth. The Nipponbare genome (IRGSP-1.0_genome, http://rapdb.dna.affrc.go.jp, accessed on 20 June 2025) was used as reference genome.

Variant calling in this study was performed using the Genome Analysis Toolkit (GATK, v 4.3.0) [31]. Vcftools (v0.1.17) was used to removes sites with >20% missing genotypes, to exclude variants with minor allele frequency < 5%, and to make sure the SNPs (single nucleotide polymorphisms) were biallelic (parameters: –max-missing 0.2, –maf 0.05, –min-alleles 2, –max-alleles 2) [32].

### 4.4. Genotyping

The linkage disequilibrium decay analysis was performed using PopLDdecay [33]. In total, 449,928 SNPs were retained after PLINK filtering according to linkage disequilibrium decay (parameters: –indep-pairwise 52 10 0.5) [34].

The genotypes from the 3000 Rice Genomes Project were downloaded from the Rice SNP-seek Database [30]. The genotype mixture of 2038 cultivars in the 3000 Rice Genomes Project and 438 cultivars in this study was performed using PLINK (v1.90b7). First, the SNP lists of 438 cultivars were extracted as common SNP lists. Then the common SNP lists were matched with the SNP lists of 2038 cultivars. The 2038 cultivars with matched SNPs were extracted and merged with the 438 cultivars using PLINK (v1.90b7) through the bmerge function. The merged SNP set went through a filter for missing rate, minor allele frequency and linkage disequilibrium decay, after which 224,361 SNPs were retained. All genotype files were imputed by beagle before being used in genomic selection [35].

### 4.5. Genomic Selection

The genomic predictions were performed using three models: two parametric models, Genomic Best Linear Unbiased Prediction (GBLUP) and Bayesian Lasso (BayesLASSO), and one non-parametric model, Light Gradient-Boosting Machine (LightGBM).

To evaluate model performance, we employed two distinct validation schemes: (1) Cross-validation (CV): A 5-fold cross-validation scheme was implemented within the set of 217 accessions with complete data for both seasons. The dataset was randomly partitioned into five equally sized subsets. For each partition, four subsets (80%) were used as the training set, and the remaining subset (20%) served as the validation set. To ensure robust estimates, this entire 5-fold CV process was repeated 20 times with different random partitions. The final prediction accuracy was calculated as the average Pearson’s correlation coefficient across all 100 validation sets (5 folds × 20 repeats). (2) Independent validation: To assess the models’ generalization ability, an independent validation was performed. The models were trained on the core set of 217 accessions, and then used to predict the phenotypes for the accessions that were unique to each season (i.e., predicting 98 accessions in the early season and 123 in the late season).

Both the GBLUP and BayesLASSO models were implemented using the BGLR package (v1.1.4) in R [36]. The linear model for both can be expressed as

(1) y=μ+Xg+ε

where y is the vector of observed phenotypes, μ is the overall mean, X is the genotype matrix (coded as 0, 1, 2 and mean-centered), g is the vector of marker effects, and ε is the vector of random residuals.

For the GBLUP model, the vector of marker effects g was assumed to follow a normal distribution, $g\sim N(0,I\sigma^{2}g)$, where $\sigma^{2}g$ is the marker effect variance.

For the BayesLASSO model, the marker effects were assigned a double-exponential (Laplace) prior, which induces sparsity. The key hyperparameter, the regularization parameter ($\lambda^{2}$), was assigned a Gamma hyperprior with a shape parameter of 1.1 and a rate parameter of 1.12 × 10^−7^. The residual variance for both models was assigned a scaled inverse Chi-squared prior with 5 degrees of freedom and a scale parameter of 3.5.

For both Bayesian models, the Markov Chain Monte Carlo (MCMC) was run for 15,000 iterations, with the first 5000 iterations discarded as burn-in. A thinning interval of 5 was used, resulting in a final set of 2000 posterior samples for inference.

The Light Gradient-Boosting Machine (LightGBM) model was implemented using the lightgbm Python package (v4.6.0) [37]. To mitigate the curse of dimensionality (*p* >> n), principal component analysis (PCA) was first performed on the genomic data using PLINK (v1.90b7). The top 335 principal components (PCs), which explained 95% of the total genetic variance, were used as input features for the model. These PC features were standardized prior to model training.

The predictive performance of all models was evaluated by calculating the Pearson’s correlation coefficient (r) between the observed phenotypic values (y) and the predicted genomic estimated breeding values (GEBVs or ŷ). The coefficient is defined as

(2) $$r=\frac{\sum_{i=1}^{n}\left(y_{i}-\bar{y}\right)({\hat{y}}_{i}-\bar{\hat{y}})}{\sqrt{\sum_{i=1}^{n}(y_{i}-\bar{y}{)}^{2}}\sqrt{\sum_{i=1}^{n}({\hat{y}}_{i}-\bar{\hat{y}}{)}^{2}}}$$

where $y_{i}$ is the observed phenotypic value for the i-th sample, ${\hat{y}}_{i}$ is the predicted value, $\bar{y}$ and $\bar{\hat{y}}$ are their respective means.

### 4.6. Genomic Heritability Estimation

To evaluate the proportion of phenotypic variance explained by genome-wide additive effects, the genomic narrow-sense heritability ($h_{g}^{2}$) was estimated for each trait in both seasons. A single-trait linear mixed model (LMM) was implemented using HIBLUP software (v1.5.3) [38]. The model is expressed as follows:

(3) y=1μ+Zg+e

where y is the vector of observed phenotypic values; μ is the overall mean; Z is the design matrix relating phenotypes to individuals; g is the vector of random additive genomic effects, assuming $g\sim N(0,G\sigma_{g}^{2})$, where G is the genomic relationship matrix (GRM) constructed using 449,928 SNPs according to VanRaden’s method [39]; and e is the vector of random residuals, assuming $e\sim N(0,I\sigma_{e}^{2})$.

The variance components, including the genomic additive variance ($\sigma_{g}^{2}$) and residual variance ($\sigma_{e}^{2}$), were estimated using the Average Information Restricted Maximum Likelihood (AI-REML) algorithm. Genomic heritability was then calculated as

(4) $$h_{g}^{2}=\frac{\sigma_{g}^{2}}{\sigma_{g}^{2}+\sigma_{e}^{2}}$$

## 5. Conclusions

In conclusion, this study demonstrates that season-specific genomic selection using parametric models is highly effective for improving lodging resistance in double-cropping rice. The decoupling of plant height and culm strength enables the breeding of a “tall-but-strong” ideotype to bypass the yield–lodging trade-off. Moreover, our results highlight that indirect selection via highly heritable proxy traits is superior to direct selection for large-scale germplasm mining. These methodologies provide a powerful tool for enhancing rice resilience and accelerating genetic improvement in intensive production environments.

## Figures and Tables

**Table 1:** Comparison of lodging-related traits between early (ES) and late (LS) seasons (*n* = 217).

Trait	Mean ± SD		*t*	df	*p*	Significance
	ES	LS				
IL3 (cm)	11.23 ± 6.58	8.13 ± 4.87	7.73	216	1.13 × 10^−15^	****
BR3 (N)	8.09 ± 4.48	16.14 ± 7.79	6.44	216	4.14 × 10^−13^	****
IL4 (cm)	19.17 ± 7.03	15.90 ± 7.32	8.66	216	2.32 × 10^−47^	****
BR4 (N)	4.84 ± 2.87	10.97 ± 5.31	18.80	216	6.31 × 10^−40^	****
IL3/PH	0.09 ± 0.04	0.08 ± 0.04	5.64	216	5.26 × 10^−8^	****
IL4/PH	0.16 ± 0.05	0.15 ± 0.06	4.56	216	8.59 × 10^−6^	****
PH (cm)	116.30 ± 22.79	107.70 ± 22.56	10.19	216	1.70 × 10^−20^	****

ES indicates early season; LS indicates late season; statistical significance was determined by paired *t*-tests. *t*, *df*, and *p* represent the *t*-statistic, degrees of freedom, and *p*-value, respectively. **** indicates *p* < 0.0001.

**Table 2:** Prediction accuracy and genomic heritability in two seasons.

Trait	Heritability	LightGBM		GBLUP		BayesLASSO	
		ES	LS	ES	LS	ES	LS
IL3 (cm)	0.5054	0.5463	0.4183	0.6583	0.5454	0.6393	0.541
BR3 (N)	0.3477	0.2019	0.5064	0.3675	0.6013	0.3644	0.61
IL4 (cm)	0.6257	0.5952	0.6084	0.7075	0.7278	0.7048	0.7225
BR4 (N)	0.2297	0.1592	0.5873	0.3317	0.6506	0.3362	0.6527
IL3/PH	0.2462	0.3908	0.4723	0.5174	0.5646	0.5169	0.554
IL4/PH	0.3724	0.5454	0.5624	0.658	0.6922	0.6541	0.6946
PH (cm)	0.7687	0.5737	0.624	0.73	0.776	0.7161	0.7511

ES indicates early season; LS indicates late season.

**Table 3:** Prediction accuracy of independent prediction in two seasons.

Trait	Heritability	LightGBM		GBLUP		BayesLASSO		SD
		ES	LS	ES	LS	ES	LS	
IL3	0.5054	0.5360	0.2247	0.6819	0.3762	0.6883	0.3682	0.1875
BR3	0.3477	0.1741	0.355	0.3453	0.4472	0.3598	0.448	0.1000
IL4	0.6257	0.6683	0.5131	0.7365	0.5859	0.7422	0.5973	0.0910
BR4	0.2297	0.2283	0.456	0.3671	0.5375	0.3923	0.5364	0.1174
IL3/PH	0.2462	0.3759	0.2647	0.6174	0.2892	0.6188	0.2797	0.1676
IL4/PH	0.3724	0.6734	0.5842	0.7468	0.5941	0.745	0.5944	0.0765
PH	0.7687	0.6560	0.5502	0.7126	0.626	0.701	0.6303	0.0590

ES indicates early season; LS indicates late season; sd indicates standard deviation.

**Table 4:** Resistance category and proportion of 3kRG.

Resistance Category	Threshold	Original Proportion
Highly Lodging-Resistant	*x* ≤ 2	31.40%
Moderately Lodging-Resistant	2 < *x* < 5	42.39%
Susceptible to Lodging	5 ≤ *x* < 7	13.74%
Extremely Susceptible to Lodging	*x* ≥ 7	12.46%

## Data Availability

The datasets of 3kRG were open-access (Rice SNP-Seek Datasets, https://snp-seek.irri.org). The genotype of 438 rice accessions and merged genotypes with 3kRG and the phenotype data used in genomic selection was uploaded in Zenodo. (https://zenodo.org, DOI: 10.5281/zenodo.17334323).

## Supplementary Materials

The following supporting information can be downloaded at https://www.mdpi.com/article/10.3390/plants15050785/s1.
